# Supplementary material for: Genetic clustering within massive Porites species complex is the primary driver of holobiont assembly
Source: PLoS One. 2025 Jul 17;20(7):e0328479. doi: 10.1371/journal.pone.0328479 (PMC12270097; doi:10.1371/journal.pone.0328479)
Supplement: S1 File — All supporting figures and tables referenced throughout the text are located in this file. (PDF) [file pone.0328479.s001.pdf]

Supplemental Information: Genetic clustering within massive *Porites* species complex is the primary driver of holobiont assembly

CB Scott, R Schott, MV Matz

Supplemental Figures

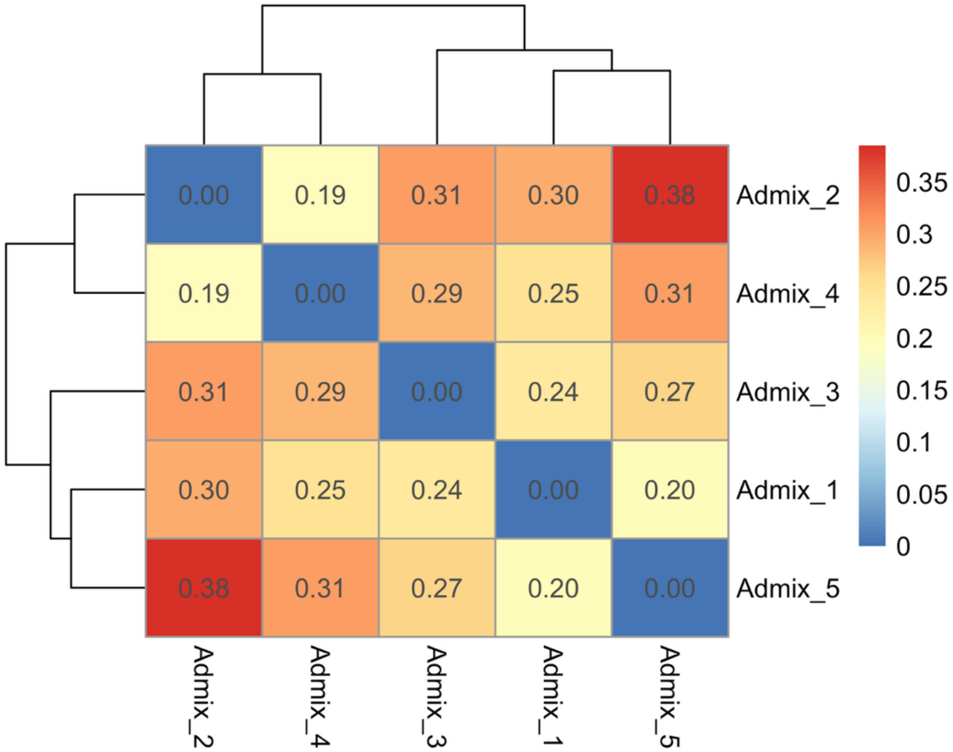

S1 Fig. Pairwise F_{ST} values between admixture groups.

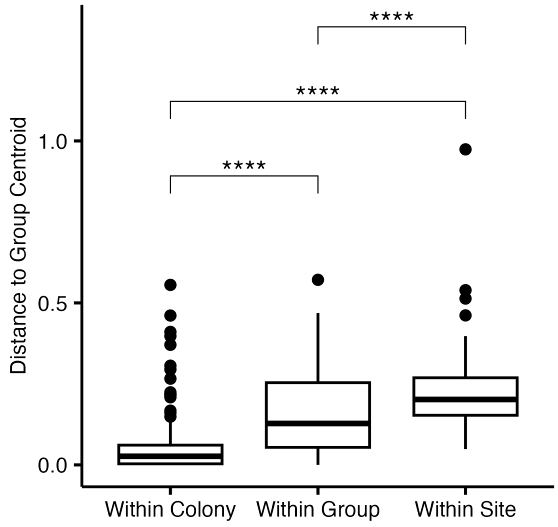

S2 Fig. Symbiodiniaceae within colony variance less than within group and within site variance. Community variance determined by distances to centroid in multivariate space. Significance determined by a pairwise Wilcox test with a significance threshold of *p=0.05*.

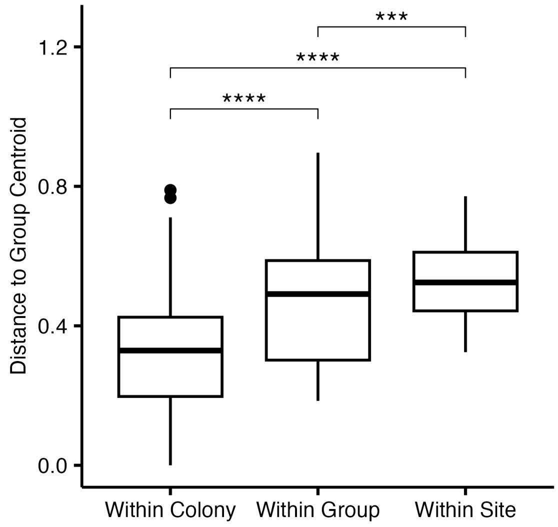

S3 Fig. Microbial within colony variance less than within group and within site variance. Community variance determined by distances to centroid in multivariate space. Significance determined by a pairwise Wilcox test with a significance threshold of *p=0.05*.

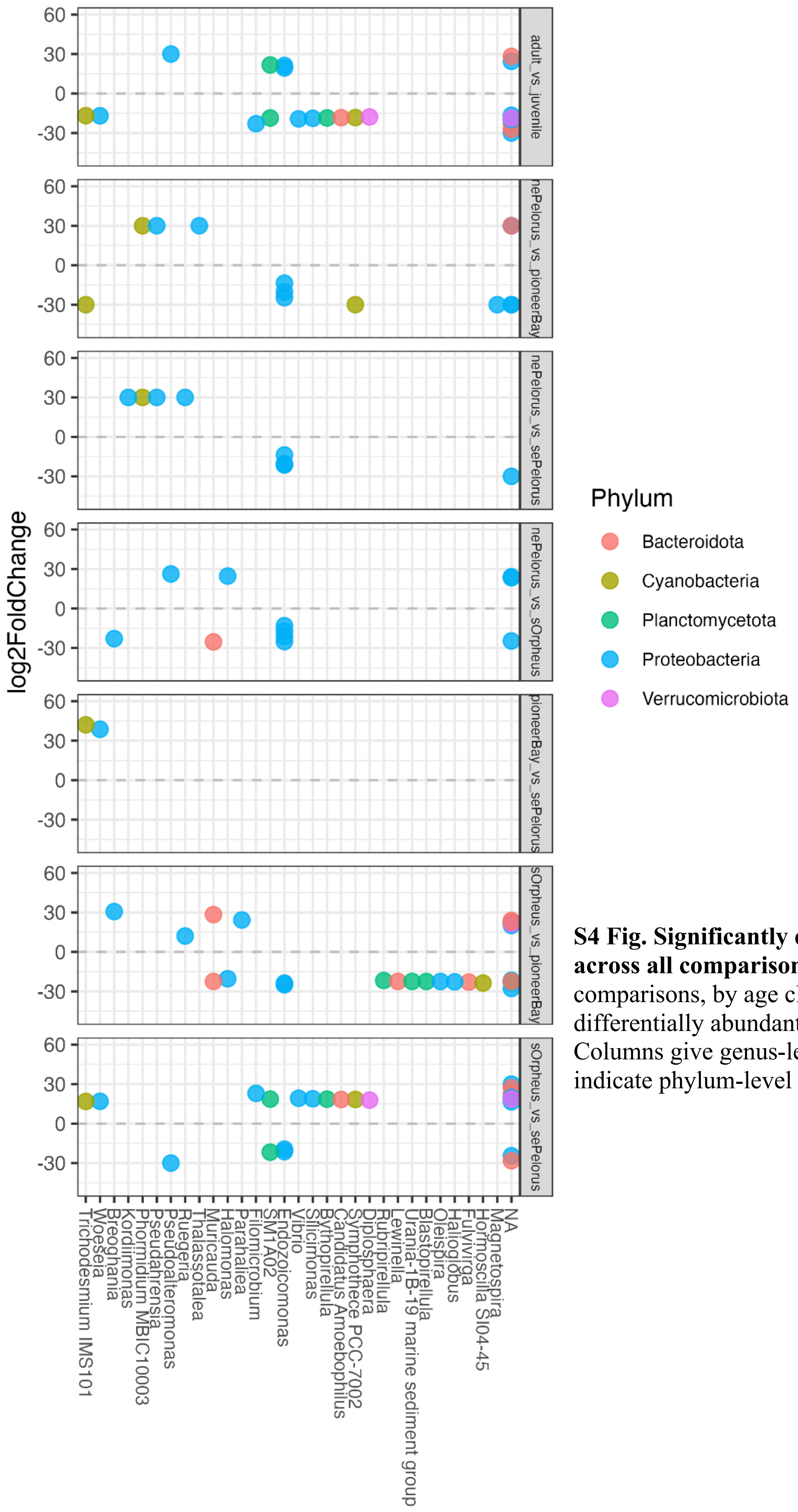

S4 Fig. Significantly differentially abundant ASVs across all comparisons. Panels give pairwise comparisons, by age class and site, of significantly differentially abundant ASVs at the α < 0.01 level. Columns give genus-level assignments, colors indicate phylum-level assignment.

Supplemental Tables

S1 Table. Symbiodiniaceae community gradient forest model importances with admixture group as a predictor. Overall gradient forest model importance (proportion variance explained) and the importance of each predictor. Higher importance corresponds to better predictive power, and an overall better model.

Predictor	Importance
AdmixGroup	0.100
Site	0.023
Size Class	0.011
Total Importance	0.135

S2 Table. Microbial community gradient forest model importances with admixture group as a predictor. Overall gradient forest model importance (proportion variance explained) and the importance of each predictor. Higher importance corresponds to better predictive power, and an overall better model.

Predictor	Importance
AdmixGroup	0.243
Site	0.155
Size Class	0.065
Total Importance	0.463
