# Supplementary material for: Genetic clustering within massive Porites species complex is the primary driver of holobiont assembly
Source: PLoS One. 2025 Jul 17;20(7):e0328479. doi: 10.1371/journal.pone.0328479 (PMC12270097; doi:10.1371/journal.pone.0328479)
Supplement: Attachment — Submitted filename: PONE-D-24-28640_Comments_to_Authors.docx [file pone.0328479.s002.docx]

**Comments to Authors for PONE-D-24-28640**

1) Summary of the research and overall impression:

The presented study provides an overview of cryptic lineages in massive *Porites* spp. on Orpheus and Pelorus Islands on the Great Barrier Reef, Australia. Cryptic diversity is large in corals in general, but specifically in massive *Porites* as other studies have found. The paper presented here expands on these previous studies and shows that the genetic structure of the host was the main driver of symbiont and microbial associations. They further disentangle the drivers reef site and size class (small, i.e. <30cm and large, i.e. >2m) on the associated microbes, where the microbiome was shaped by reef site and size class and the symbiont community was unaffected by both. In light of growing restauration efforts and other techniques to mitigate the impact of climate change on coral reefs, this study adds onto the relevant literature identifying cryptic diversity in corals and provides a framework for understanding holobiont flexibility in light of environmental change. The paper is well-written, the methodology is thorough and the conclusions drawn are comprehensible. I would advise to accept this manuscript with minor changes.

2) Minor issues

**General comments:**

You should use a grammar checking tool. I have underlined some of the issues in the following, but not all. The issues are minor, but sometimes you are missing a “the” or an “of”. Further, make sure to decide on either “2bRAD” or “2b-RAD” and use it throughout. And, often there is a space between “>” and the following value, but sometimes there isn’t. Decide on one and keep the scheme throughout. The same goes for units, please add a space between the value and its corresponding unit in all instances. Lastly, please check that *Porites* is in italic in all instances. I added some below but did not comment on each case.

**Abstract**

I would specify the size classes of *Porites* here already. E.g. “[...] of massive Porites sampled across two size classes (small, <30cm and large, >2m) [...]”. I would also put “small” first and then large and not the other way around since the Introduction as well as Fig. 1 follow the same trend.

**Introduction**

General comment: You make the case that long-term studies are lacking to disentangle shifts in the microbial communities of coral holobionts, but it is difficult to make your case without confirming whether the juveniles are direct offspring of the large colonies. In terms of symbionts it is possible, since you show stable associations with specific symbiont types and this is generally the case in many corals, but the microbiome is a different story. So I would suggest to add a couple of sentences either here or in the discussion about the limitations of such inferences.

L. 92: “Here, we compared host-associated communities of massive Porites across size classes.”

LL.99f: Since you specify the year in which your samples were taken, I would add the season as well. Otherwise I would leave out the year as it is technically not relevant in the introduction. You specify it in your methods, which is where it is important.

LL.99f: I would write out the names of each site and add the abbreviation in brackets behind it. E.g. “North East and South East Pelorus (NE and SE Pelorus, respectively) [...] and South Orpheus (S Orpheus) is also a shallow nearshore site [...]”

**Methods**

LL. 133-134: “Samples were collected from four sites around the two islands [...]”

ll. 159ff: I would give a reasoning for the genomes you used. E.g. why did you choose *P. lutea* as a reference? If massive *Porites* in Australia have not been identified to species level would it have been beneficial to combine multiple *Porites* reference genomes into your reference? I mainly ask since *P. lobata* is generally used for *Porites* sp. and the reference genome published by Noel et al. (2023) has a higher completeness and continuity than *P. lutea*.

LL. 166ff: You use “at this point” twice, please find a synonym for one of the instances.

LL. 181: A reference is missing for realSFS.

LL. 183ff: Make sure to use the same mode of reporting for the models used for your PERMANOVA analyses. In the first paragraph you specify it in brackets, in the second paragraph you specify it within the sentence (l. 192). The same goes for ll. 248-250 and on page 7.

L. 253: PCoA

**Results**

L. 293: Please remove the space between “sub clustering”.

L. 313: “The vast majority [...] were identified as C15”

L. 344: Please remove “find”.

**Discussion**

L. 361: Please put *Porites* in italic.

L. 367: Please provide some references for this statement.

L. 373: I am unsure that “host” is the correct verb here. Further, Grupstra et al. (2024) did not determine the definite taxonomy of the cryptic lineages identified. Therefore, please rephrase it along the lines of “This aligns with recent work showing that cryptic lineages of massive *Porites* at environmentally distinct sites in Palau harbor diverging holobiont strategies”

L. 375: “genetic subclusters”

LL. 378-379: I would add here that the differences between genetic clusters is driven by subgeneric partitioning *within* C15 and to a lesser extent by symbionts of potentially the same genus or other genera that weren’t classified.

Ll. 396ff: Please use commas instead of “;”

L. 403: There is a “.” missing

LL. 406ff: I would expand a bit more on the differences in *Endozoicomonas* abundance between the sites. Since there is quite some literature on *Endozoicomonas* now, it should be mentioned briefly here.

**Conclusion**

LL. 421f: I would write a more comprehensive first sentence to conclude the manuscript. E.g. “We demonstrated that cryptic genetic structure was the most important factor in determining both coral-associated Symbiodiniaceae and microbial communities in massive *Porites* at Orpheus and Pelorus Islands, Australia.”

L. 424: I would add “[...] than the association with Symbiodiniaceae”

**Figures**

Fig.1: The legend of Figure 1 needs to be checked for grammar. E.g. “These islands are located on the inner Great Barrier Reef [...]”.

Fig. 2: “(a) Proportion of individuals from each admixture group at each site. (b) Admixture plot [...]”

Fig. 3: I would say “[...] exists among *Porites* spp. admixture groups”.

Fig. 4: Please edit the spelling within the figure to “ASVs” instead of “ASVS”. Further, for comprehension I would add the description of D4 ASVs to the figure legend.

Fig. 5: Please put *Porites* in italic.

Fig. 6: You show the admixture group colours in Fig. 3 but I would add them here too. I think it would benefit the figure to be able to understand it without the help of another figure.

Table 2 & 3: To make it the same across all Tables please add the “0” to all values below 1.

Table 3: Throughout the manuscript you speak of size class rather than age. Please change age to size class here.
