# Supplementary material for: Genetic clustering within massive Porites species complex is the primary driver of holobiont assembly
Source: PLoS One. 2025 Jul 17;20(7):e0328479. doi: 10.1371/journal.pone.0328479 (PMC12270097; doi:10.1371/journal.pone.0328479)
Supplement: Attachment — Submitted filename: Response to Reviewers.docx [file pone.0328479.s004.docx]

To the Editor(s) at *PLOS ONE:*

Thank you for securing detailed reviews for our manuscript, “Cryptic genetic structure of the coral host is the primary driver of holobiont assembly in massive *Porites,”* for publication in *PLOS ONE*. We appreciate the insightful comments left by the reviewers, which have improved the clarity and interpretation of our results. We have incorporated most of the suggestions provided by the reviewers, which are highlighted within the manuscript. Please see below, in blue, for a point-by-point response to the reviewers’ comments. We note that Reviewer 1 suggested many small grammatical corrections which have improved the readability of our manuscript. Rather than respond to each of these suggestions individually below, we have simply made all the requested grammatical changes in the manuscript. We additionally note that while all content changes have been noted in the tracked changes version of the manuscript, we further edited style only in the clean version of the manuscript submitted to better reflect *PLOS ONE’s* style guidelines.

Additionally, in response to editor comments from *PLOS ONE*:

1. We have updated our funding disclosure statement and edited our acknowledgements to remove funding information. This is reflected in the cover page to our paper. The new funding statement now reads:

This research was supported by NSF DGE 2137420 to C.B.S, NSF grant IOS-1755277 to M. V. M., funding from the University of Texas Department of Integrative Biology to C.B.S, and funding from the International Women’s Fishing Association to C.B.S. The funders had no role in the study design, data collection and analysis, decision to publish, or preparation of the manuscript.

The new acknowledgements statement reads:

The data analysis for this manuscript has been performed using facilities of the Texas Advanced Computing Center (TACC). We thank Kristina Black, Greg Torda, and JP Rippe for their help collecting these samples and their camaraderie in the field and the staff of Orpheus Island Research Station for supporting the logistics of this project.

2. We have published our data in open access repositories. All sequencing data can now be found at PRJNA1048506 and processing scripts are on GitHub at https://github.com/cb-scott/PoritesHolobiont_Final and on Zenodo at 10.5281/zenodo.15565326. The data availability statement has been updated to reflect this.
3. Figures 1 & 2 contain satellite image data. We created these maps ourselves from raw geospatial files provided by the Great Barrier Marine Park Authority (<https://catalogue.eatlas.org.au/geonetwork/srv/eng/catalog.search#/metadata/ac8e8e4f-fc0e-4a01-9c3d-f27e4a8fac3c>). This data is protected by a Creative Commons – Attribution 4.0 International license, which if we understand correctly, enables the redistribution of the material in any medium or format for any purpose. Please let us know if this is not the case. To clarify this, we have included a reference to this raw dataset in our methods and figure captions. The map of Australia in Figure 1 was sourced from the ‘maps’ package data in R. This data comes from the Natural Earth Data project (<https://www.naturalearthdata.com/>), which is a public domain map dataset.

**Reviewer 1**

Reviewer #1: The presented study provides an overview of cryptic lineages in massive Porites spp. on Orpheus and Pelorus Islands on the Great Barrier Reef, Australia. Cryptic diversity is large in corals in general, but specifically in massive Porites as other studies have found. The paper presented here expands on these previous studies and shows that the genetic structure of the host was the main driver of symbiont and microbial associations. They further disentangle the drivers reef site and size class (small, i.e. <30cm and large, i.e. >2m) on the associated microbes, where the microbiome was shaped by reef site and size class and the symbiont community was unaffected by both. In light of growing restauration efforts and other techniques to mitigate the impact of climate change on coral reefs, this study adds onto the relevant literature identifying cryptic diversity in corals and provides a framework for understanding holobiont flexibility in light of environmental change. The paper is well-written, the methodology is thorough, and the conclusions drawn are comprehensible. I would advise to accept this manuscript with minor changes.

**General comments:**

You should use a grammar checking tool. I have underlined some of the issues in the following, but not all. The issues are minor, but sometimes you are missing a “the” or an “of”. Further, make sure to decide on either “2bRAD” or “2b-RAD” and use it throughout. And, often there is a space between “>” and the following value, but sometimes there isn’t. Decide on one and keep the scheme throughout. The same goes for units, please add a space between the value and its corresponding unit in all instances. Lastly, please check that *Porites* is in italic in all instances. I added some below but did not comment on each case.

The reviewer’s attention to detail is appreciated, we have made the requested changes throughout and checked for consistency. The reviewer also suggested many minor grammatical and phrasing changes in their comments. We have made all of the suggested changes throughout the manuscript (and have highlighted them in a tracked-changes version) but have not responded to each individual grammatical comment in their review.

**Introduction**

General comment: You make the case that long-term studies are lacking to disentangle shifts in the microbial communities of coral holobionts, but it is difficult to make your case without confirming whether the juveniles are direct offspring of the large colonies. In terms of symbionts it is possible, since you show stable associations with specific symbiont types and this is generally the case in many corals, but the microbiome is a different story. So I would suggest to add a couple of sentences either here or in the discussion about the limitations of such inferences.

We have added some text to the discussion about the limitation of such inferences and some recommendations for future studies on lines 318- 323 of the revised manuscript:

Our study highlights that studies of coral holobiont dynamics must account for host genetic background, as fine-scale differences may be obscured by lineage-specific communities. However, our study did not confirm whether small *Porites* on the reef are the direct descendants of concurrent massive individuals. Future studies evaluating the role age and environmental drivers in structuring the holobiont community would be most robust if relatedness between individuals was controlled for (e.g., through the out planting of offspring with known parents).

**Methods**

ll. 159ff: I would give a reasoning for the genomes you used. E.g. why did you choose *P. lutea* as a reference? If massive *Porites* in Australia have not been identified to species level would it have been beneficial to combine multiple *Porites* reference genomes into your reference? I mainly ask since *P. lobata* is generally used for *Porites* sp. and the reference genome published by Noel et al. (2023) has a higher completeness and continuity than *P. lutea*.

We appreciate that the reviewer has highlighted a more complete reference. Quite simply, we did not use this reference as it was not yet published when we began our analysis. We remapped our data to the updated reference and evaluated initial population genetic structure. This yielded qualitatively the same placement of samples in PCA space.

Given the similarity of sample placement, we retained our analysis using the original genome.

**Discussion**

LL. 378-379: I would add here that the differences between genetic clusters is driven by subgeneric partitioning *within* C15 and to a lesser extent by symbionts of potentially the same genus or other genera that weren’t classified.

The reviewer is correct in their interpretation of our results – the patterns we observed could have been due to partitioning within C15, by the differences in background symbiont communities, or by differences in unclassified taxa. We have changed our discussion text to read on lines 300 – 302 of the revised manuscript:

All coral colonies were dominated by symbiont strain C15, so differences between genetic subclusters are being driven by subgeneric partitioning *within* C15, background strains, or, to a lesser extent, by symbionts of potentially the same genus or other genera that weren’t classified (Figure 4).

LL. 406ff: I would expand a bit more on the differences in *Endozoicomonas* abundance between the sites. Since there is quite some literature on *Endozoicomonas* now, it should be mentioned briefly here.

The reviewer’s comments on the body of literature on *Endozoicomonas* are well merited. We had initially hesitated to make too strong of claims about *Endozoicomonas* function as we have only classified diversity to the genus level and there is significant debate about the role of specific *Endozoicomonas* species. We have included a more in-depth discussion of this evolving literature and possible limitations on lines 328—339 of the revised manuscript, in lieu of the original text on lines 406 - 410:

Work quantifying *Endozoicomonas* at the bacterial lineage level has shown that *Endozoicomonas* strains are often geographically distinct in *Porites* corals, and some strains may have genes indicating their involvement in the sulfur cycle (Hochart et al., 2023). This suggests that the between-site differences we recovered may reflect functional, strain-level differences related to the local environment. However, we were only able to classify *Endozoicomonas* OTUs to the genus level with our data and massive diversity exists within the genus. It is clear that *Endozoicomonas* species can span the mutualism to parasitism continuum (Pogoreutz & Ziegler, 2024). While some species of *Endozoicomonas* may be beneficial microbial symbionts modulating the environment for their coral hosts, others may be parasites of marine taxa or opportunistic colonizers from the environment (Peixoto et al., 2017; Tandon et al., 2022; Katharios et al., 2015). To make stronger claims about the role of *Endozoicomonas* to the *Porites* holobiont in different environments, future work should generate metagenomic datasets to assess species and functional diversity while controlling for host genetic background.

**Figures**

Fig. 4: Please edit the spelling within the figure to “ASVs” instead of “ASVS”. Further, for comprehension I would add the description of D4 ASVs to the figure legend.

In response to Reviewer 2, we have included an updated version of this figure which also corrects this error.

Fig. 6: You show the admixture group colours in Fig. 3 but I would add them here too. I think it would benefit the figure to be able to understand it without the help of another figure.

We have added a legend to this figure, corresponding to the admixture group colors defined in Figures 3, 4, and 5.

**Reviewer 2**

Reviewer #2: The ms presented by Scott et al. is a well written and data rich study that investigates factors responsible for prokaryotic and algal microbiome composition of Porites spp. corals. There has been relatively little focus to dissect detailed microbial and algal strain diversity in relationship to fine scale host genetics. In that respect, the study is timely and interesting to the coral community and beyond. The methodology looks sound to me, the presentations are generally clear.

My main concern is the notion of “cryptic genetic structure” that already appears in the title. My understanding is that the authors study a species complex, genus Porites, where the existence of several species is an acknowledged fact. Being not a coral expert, I assume that these species are morphologically so plastic and /or similar that non-genetic taxonomy will not work. This is also rightly acknowledged by the authors in line 366.

By means of Rad-seq /SNP calling they are able to resolve them to species level which is an interesting finding in and among itself. If so, it is rather (the difficult to determine) taxonomic affiliation to a species within a genus that determines the composition of algal and prokaryotic symbionts. As such, the entire data set would also be interesting data worthwhile to report, but with a changed interpretation /implication. As it stands now, the title implies (to me) within-species genetic structure.

The reviewer is correct that we cannot formally distinguish between nominal (very hard to identify) Porites species and cryptic lineages within each species. We agree that it is confusing that we call our genetic clusters “cryptic lineages”, and that some wording needs to be changed.

To correct this confusion, we have changed “cryptic lineages” to “genetic clusters” throughout and have added the following text into the Discussion to highlight this:

Lines 284-292: Likely, the distinct admixture groups recovered reflect the sampling of multiple species of *Porites* spp., as they are morphologically plastic and notoriously difficult to identify in the field. Some of the genetic clusters that we have identified may correspond to different nominal species of Porites, while others may correspond to cryptic lineages within these species. We could not discriminate between these possibilities for the lack of samples and expertise to analyze *Porites* spp. morphology. We therefore chose to talk about “genetic clusters” rather than species or cryptic lineages (which are supposed to belong to the same nominal species).

We have additionally changed the wording of the title to reflect this shift in interpretation:

**Genetic clustering within massive *Porites* species complex is the primary driver of holobiont assembly**

Along the same lines, in order to benchmark the differences among the host genetic clusters (aka cryptic species?) it would be great to quantify /date the divergence among coral hosts, maybe with some mitochondrial genetic clock? This way, the data would become comparable to other such studies that look at the effects of host genotypic /species affiliation for microbial community assembly.

We agree with the reviewer that quantifying the divergence among coral hosts with a mitochondrial clock would increase this study’s comparability with existing work. However, with our existing 2bRAD data, we only recovered one overlapping site between samples which mapped to the *Porites lutea* mitochondrial genome, which was insufficient for downstream analysis. In an attempt to quantify divergence, we calculated pairwise F_ST_ between genetic clusters. These calculations are included in Supplemental Figure 1. Additionally, we have visualized the pairwise F_ST_ values between genetic clusters/admixture groups as an unrooted tree in panel ‘b’ of Figure 3.

Finally, can any specific inference be made as to the level of species-affiliated genomic divergence vs. within species genetic structure, based on the data of the host? I note that there are two levels of clustering that the authors describe, but this is not picked up later in the ms. Maybe the combined sample size is too low here?

We appreciate the reviewer’s concerns about the two levels of genetic clustering. Upon revisiting this topic (combined with the feedback below on the admixture plot), we have decided to temper our claim about genetic clustering *within* the admixture groups. This comment prompted us to reevaluate our clustering methodology, and we realized that clustering outcomes beyond the five admixture clusters are sensitive to methods and parameters chosen. Therefore, we decided to fall back to the five admixture clusters to represent the genetic structure in our data. We had already run this analysis in parallel with the original analysis that we presented and had recovered qualitatively the same result (in the supplemental figures and tables). We have modified the manuscript to instead center the admixture group-based analysis in the main text and figures, as we feel like the determination of the five primary genetic clusters is less subjective. We reran all analyses and remade all the figures. The results remain essentially the same as before.

The reviewer was also correct in their assessment that there was insufficient sample size at the finer scale of clustering to make meaningful biological conclusions. We attempted to run our analysis for the Symbiodiniaceae and microbial communities within each genetic cluster as well, but the sample size was too low in most cases to recover results from either PERMANOVA or gradient forest methodology.

Further suggestions, in the order of appearance

L58: the study results do not really present anything on the role of the associated microbial community for holobiont adaptation /resilience, so a different intro lead would be more appropriate for the paper

We agree that this lead was out of place with the rest of the paper. This paragraph is meant to be an introduction for the reader to the importance of the coral holobiont and why our work (and prior studies) has focused on it. We have edited the paper led to instead read on Lines 59-62 of the revised manuscript:

~~The rapid rate of climate change poses an immediate threat to coral reefs globally.~~ ~~Environmental change acts on the entire coral holobiont,~~ The coral holobiont, or the collective unit of the coral animal, its intracellular algal symbionts, and its associated microbial community, determines how coral reefs respond to environmental change (Goulet et al., 2020).

Additionally, in response to this suggestion, we have made minor changes throughout the first two paragraphs in the introduction to place less emphasis on the adaptive capacity of the holobiont and highlight better what is unknown about coral holobiont assembly:

Removed text on lines 65 – 67: Thus, corals may keep pace with climate change in one of two ways – through the evolution of the coral host animal and/or through holobiont community reassembly (potentially driven by host evolution).

Lines 64 – 65: However, the underlying mechanisms which determine coral holobiont assembly are still not fully understood.

L88: This reads awkward, as the data presented are also not long term, but deal with a single survey

We have changed the phrasing to instead read in the revised manuscript:

Lines 81 – 82: While this is possible, most studies fail to integrate ~~long-term (e.g., greater than 5 year) temporal data~~ individual-level genotyping, size class, and site data to disentangle these effects.

L99: an overview on the “site * colony size * replicate sample” combinations would be nice, and also the absolute spatial distances at all scales.

We have edited the intro to this paragraph to read:

Lines 84 – 95: Here, we compared host-associated communities of massive *Porites* across size classes (small, < 30 cm and massive, >2 m), sites (spanning 13 km), and at five genetic clusters.

Fig 4. Here, the community composition patterns look like rather uniform among host groups. This might partly be a result of the “binning” of the color coding. But this is confusing as the diagram in its present form suggests hardly any structure. This figure should convey as much qualitative differentiation as possible to explain the clear MDS clustering in later analyses.

As a suggestion, is it possible to look at a heat map for each individual sample clustered (i) by microbial /algal composition (ii) by host genetics? Such a diagram could convey everyting at a glance.

We agree that the community profile figures (both Figures 4 and 5) could better highlight the diversity present in our samples. We have included updated figures in the manuscript in line with the reviewer’s suggestions, presenting our community data as a clustered heatmap rather than a stacked bar plot. While these figures qualitatively show the same information, the role that admixture group plays in structuring both communities are visually more pronounced.

It is worth noting that for the Symbiodiniaceae community, there is little variation between colonies at the genus level. Most are dominated by the same strain of C15, which can be clearly seen on our new Figure 4. Reviewer 1 also commented on this, and we have included the following text in the discussion on lines 300 – 302 in the revised manuscript to make this pattern clearer:

All coral colonies were dominated by symbiont strain C15, so differences between genetic subclusters are being driven by subgeneric partitioning within C15, background strains, or, to a lesser extent, by symbionts of potentially the same genus or other genera that weren’t classified (Figure 4).

L363: I would present a STRUCTURE graph also with 10 clusters, as you repeatedly refer to that optimal cluster number rather than 5 (as in Fig 2).

As highlighted above in our letter, the reviewer’s feedback prompted us to revisit our clustering methodology. Due to this, we have revised our methodology and removed our claims that 10 clusters is the optimum number. Ultimately, we think that the 5 clusters returned by the initial admixture analysis is the most robust categorization of our genetic data and have presented this as the main result. Thus, we have not included a STRUCTURE graph with 10 genetic clusters.
